# Metagenomic mining of environmental resistomes: Comparative performance of deep learning and alignment‐based annotation methods

**DOI:** 10.1002/imo2.70107

**Published:** 2026-06-09

**Authors:** Joeselle M. Serrana, Francisco J. A. Nascimento, Malte Posselt

**Affiliations:** ^1^ Stockholm University Center for Circular and Sustainable Systems (SUCCeSS) Stockholm University Stockholm Sweden; ^2^ Department of Environmental Science (ACES) Stockholm University Stockholm Sweden; ^3^ Department of Ecology, Environment, and Plant Sciences (DEEP) Stockholm University Stockholm Sweden

## Abstract

Antimicrobial resistance (AMR) in marine environments poses intricate challenges due to the ecological complexity of microbial communities and environmental gradients. This study compares various antimicrobial resistance gene (ARG) annotation tools and databases, focusing on deep learning approaches versus traditional alignment‐based methods, to evaluate their effectiveness in profiling the resistome of a complex marine ecosystem. Our report not only highlights the utility of deep learning methods for environmental applications but also proposes pathways for future improvements that can bridge the gap between comprehensive detection and clinically relevant risk interpretation.

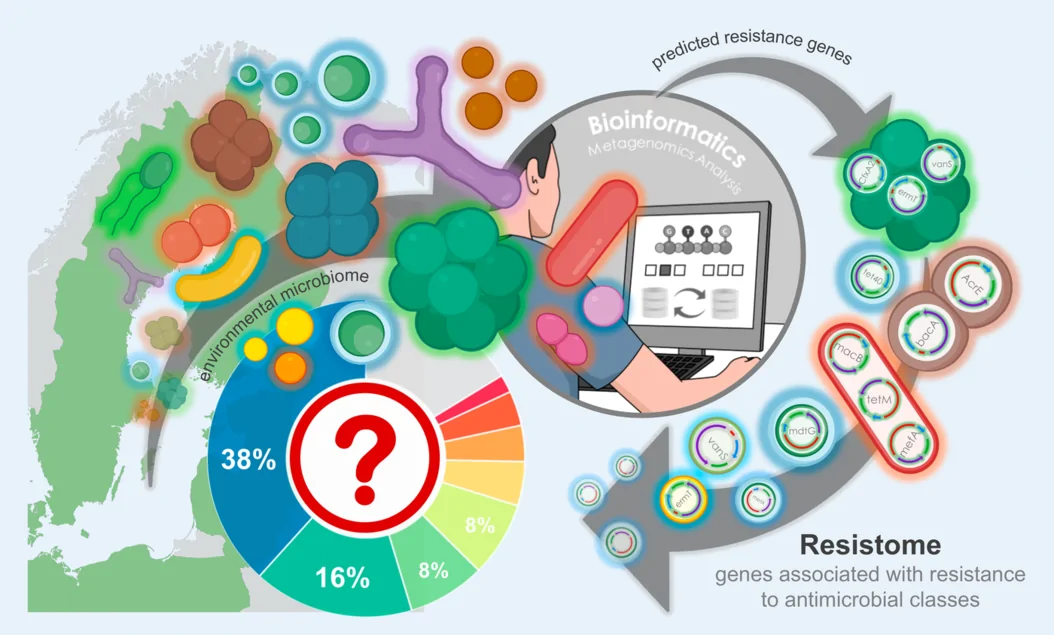

To the editor,

Antimicrobial resistance (AMR) is a major global health threat caused by the spread of resistant pathogens and genes among humans, animals, and the environment, making it a complex issue to address [1, 2, 3]. Marine ecosystems are particularly important yet challenging to understand in the context of AMR due to their diverse microbiomes and variable environmental conditions [4, 5]. Although significant progress has been made in bioinformatics analysis, selecting the right antimicrobial resistance gene (ARG) database or annotation method remains challenging due to notable differences in structural design, curation approach, annotation levels, and the extent of resistance gene coverage [6]. Moreover, most ARG annotation methods rely on alignment‐based approaches using curated databases, for example, CARD, ResFinder, MegaRes, and SARG [7]. These approaches use best‐hit or BLAST searches and have proven effective in clinical settings [8], but they can be limited in detecting ARGs in complex environmental samples, where genes may have low similarity to reference sequences and could include novel or cryptic variants [6, 7, 9]. Deep learning‐based approaches overcome some of these limitations by using pattern recognition techniques to identify ARGs even when sequence similarities fall below traditional thresholds. For example, DeepARG relies on a model trained on multiple reference databases (e.g., CARD, ARDB, and UniProt) and may reduce false negatives while improving robustness in detecting ARG diversity, especially in complex environmental samples [10].

Several studies have demonstrated that the selection of an annotation database or tool can significantly impact downstream analyses [11, 12]. However, there is a lack of assessments of its impact on the estimation of ARG diversity and composition, as well as its relationship with environmental factors. Additionally, differences in classification schemes, where some databases group multidrug resistance broadly while others distinguish antibiotic classes into separate groups, can change the perceived dominance of particular ARG types in a sample. Given these factors, a comparative analysis is necessary to evaluate how different ARG annotation methods, for example, deep learning‐based approaches and traditional alignment‑based pipelines, influence resistome characterization and downstream ecological interpretation in environmental contexts.

Here, we examined whether different ARG databases and annotation tools affect the resistome profile derived from a complex marine environment (i.e., the Baltic Sea). Importantly, this study does not aim to identify the most accurate ARG annotation tool but rather to quantitatively assess how commonly used annotation pipelines, when applied under standard, real‑world conditions, may lead to different ecological interpretations. Hence, we compared several annotation approaches and databases to evaluate ARG annotation, diversity, and environmental associations (Table S1). We focused on the performance of DeepARG [10], a deep learning‐based tool, compared to traditional alignment‐based approaches, that is, AMR++ v3.0 [13] and BLAST‐based matching to CARD v4.0 [14], ResFinder v4.0 [15], ResFinder FG v2.0 [16], MegaRes v3.0 [13], SARG v3.2.1‐S [17], and PanRes v1.0 [18]. For all contig‑based annotation workflows (i.e., DeepARG, CARD, ResFinder, ResFinderFG, SARG, and MegaRes), gene abundances were normalized using reads per kilobase per million mapped reads (RPKM) to account for gene length and sequencing depth. It is important to note that the compared pipelines operate on different input data types (i.e., read‑based annotation for AMR++ vs. contig‑based annotation). These differences reflect typical real‑world usage rather than a controlled benchmarking design. Assembly processes inherently filter out low‑coverage or fragmented sequences, potentially removing rare ARGs that would otherwise be detectable in read‑based workflows. Hence, our comparisons do not constitute a strict performance evaluation but rather illustrate how pipeline design may influence downstream ecological interpretations. Below, we present a thorough evaluation and discussion of both the quantitative results and the environmental associations of the resistome profiles obtained through these different methods.

## 1 COMPARATIVE PERFORMANCE OF AMR PREDICTION TOOLS AND RESISTOME PROFILE DIVERSITY CORRELATIONS

In terms of raw annotation counts under our current parameterization and inputs, DeepARG identified the highest number of ARG‐related annotations from the Baltic Sea metagenomes, with 2042 annotations representing 378 unique ARGs (Figure 1A) belonging to 26 ARG types and 378 subtypes (i.e., ARGs and resistance‐associated genes) (Figure 1B, Figure S1 and Table S2), where unique ARGs refer to distinct ARG gene families or subtypes as defined by the tool's native annotation scheme. This contrasts with the alignment‐based approaches. For example, PanRes identified 1656 genes associated with AMR, whereas both ResFinder (with an 80% identity threshold) and ResFinder FG detected only 11 and 7 annotations, respectively. The categories are not harmonized across databases (e.g., ARO terms) and therefore represent database‑specific ARG groupings rather than standardized sequence clusters. However, the discrepancy in annotation counts among the methods underscores that the choice of database and the underlying classification algorithm may affect quantitative estimates of ARG abundance and diversity. These differences align with earlier reports indicating that ARG annotations are subject to sampling biases due to the content and update frequency of each reference dataset [10, 11, 12, 19]. Still, we note that the higher annotation counts from DeepARG should not be interpreted as sole evidence of superior performance. Without method‑agnostic accuracy benchmarks, increased detection may reflect higher sensitivity, elevated false‑positive rates, or both. As such, our observed differences in annotation counts represent methodological differences rather than validated accuracy.

**Figure 1:**
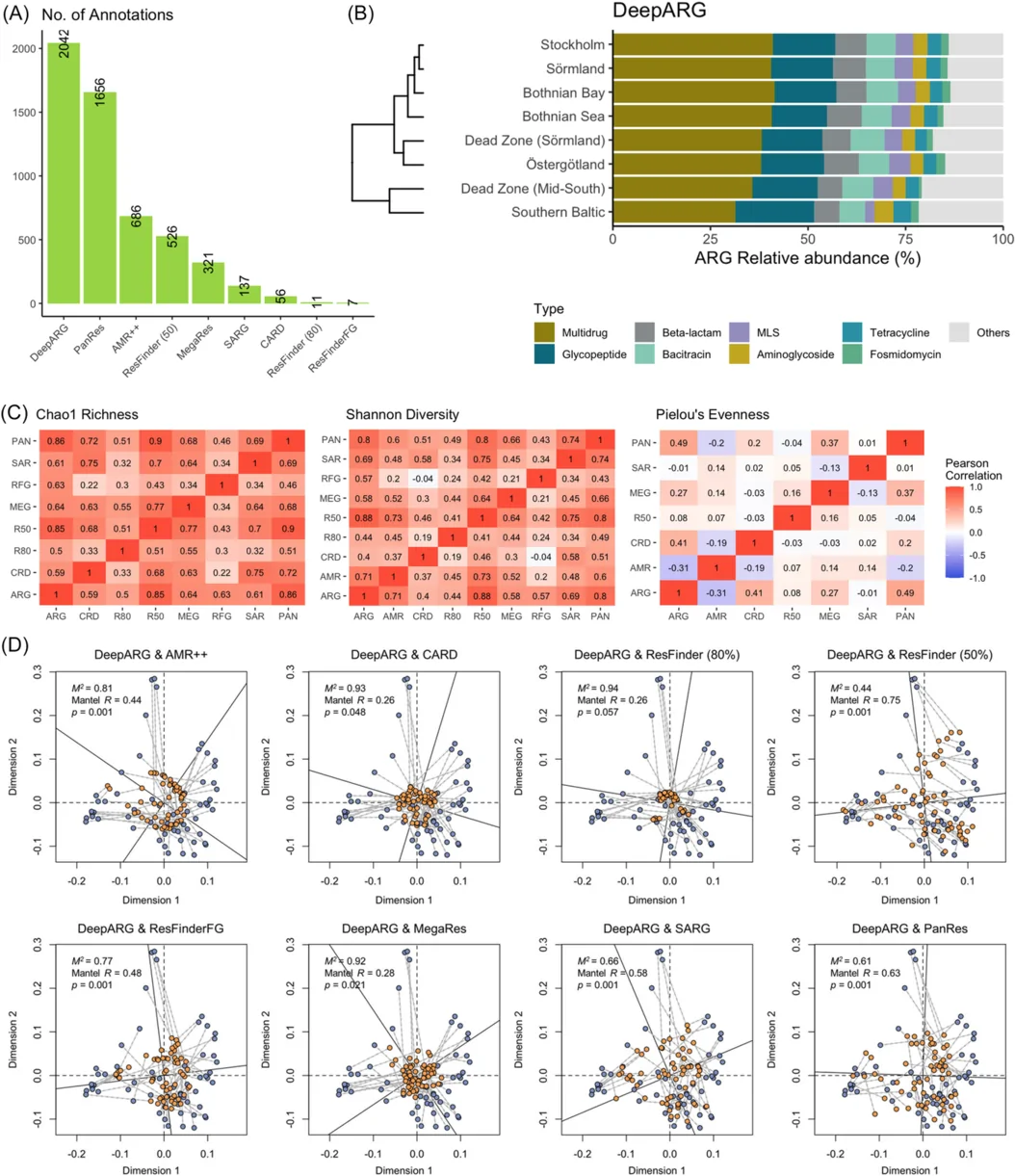
Comparison of the antimicrobial resistance (AMR) prediction tools. (A) Bar plots of the number of annotations per method/database. (B) Relative abundance of AMR annotation by type based on DeepARG prediction (detailed visualization in Figure S1). (C) Correlation heatmaps of the Chao1 richness, Shannon diversity, and Pielou's evenness of each method. ARG indicates the DeepARG profile, AMR for AMR++, CRD for CARD, R80 and R50 for ResFinder, RFG for ResFinder FG, MEG for MegaRes, SAR for SARG, and PAN for PanRes v1.0. (D) Mantel tests between DeepARG annotations and the other methods. Blue circles represent samples with DeepARG annotations, and the orange circles represent the other method.

Alignment‐based tools and highly curated databases, e.g., CARD and ResFinder, provide conservative estimates of ARG profiles, emphasizing clinically relevant and well‐characterized resistance determinants [16]. This level of specificity is valuable when the goal is to minimize false positives and focus on clinically relevant resistance. On the other hand, the machine learning‐based tool, i.e., DeepARG, excelled at capturing a broader spectrum of resistance‐associated sequences, including putative or novel ARGs that lie outside strictly defined similarity thresholds. This increased sensitivity is particularly valuable for exploratory environmental studies where the resistome is expected to be highly diverse and influenced by non‐clinical factors [10].

Additionally, another factor contributing to differences in detection between methods is the varying classification schemes and thresholds used by each database. For example, ResFinder (with a strict 80% identity threshold) returned a low number of annotations (11 annotations), but the number increased when the threshold was lowered to 50% (526 annotations). This suggests that altering sequence similarity thresholds can lead to substantial variability in ARG detection, underscoring the importance of employing multiple complementary approaches to validate results [20]. The increase in annotations at 50% identity reflects the expected trade‑off between sensitivity and precision, and values near this threshold may include spurious matches, particularly for short sequences. Accordingly, these results are presented solely as a sensitivity analysis. We note that the 50% identity threshold was included to illustrate the effect of relaxing similarity cutoffs, and because identity values near this threshold may approach random similarity for short peptides, these results are mainly interpreted not as biologically definitive but rather as an exploration of parameter sensitivity. Hence, the strong sensitivity of ResFinder to identity thresholds underscores the importance of balancing sensitivity and precision in ARG annotation. Lower thresholds may inflate false positives, and without method‑agnostic benchmarking, precision cannot be reliably assessed.

Moreover, the differences in classification categories, for example, the multidrug resistance category being most common in DeepARG versus the drug and biocide resistance category reported by AMR++ (Figure S1), further highlight that a single annotation method may not fully capture the complexity of the resistome. These differences reflect not only database‐specific ontologies but also the broader challenge of accurately capturing the multifaceted nature of environmental resistomes. Nonetheless, it is also important to note that DeepARG incorporates UniProt‑derived sequences as part of its curated training and validation dataset. While these sequences contribute to the model's ability to detect divergent or low‑similarity ARG candidates, they do not represent experimentally confirmed resistance determinants. As such, UniProt‑derived annotations should be interpreted as putative ARGs, reflecting the model's broader annotation space rather than independently validated functional evidence.

Further analysis involved evaluating how each annotation method influenced the ecological structure and diversity of the resistome profile. After calculating various diversity indices, we conducted Pearson correlation analyses to compare richness (Chao1), diversity (Shannon), and evenness (Pielou's evenness) across methods (Figure 1C). DeepARG's diversity estimates were found to be correlated with those derived from ResFinder (using a relaxed 50% identity threshold) and PanRes with regard to both the Chao1 and Shannon indices, and specifically with PanRes for Pielou's evenness index. Additionally, a Procrustes analysis was performed to compare the compositional profiles (based on Bray–Curtis dissimilarity) of each annotation method against DeepARG (Figure 1D). ResFinder (at a 50% identity threshold) (Mantel *R* = 0.75, *p* = 0.001) and PanRes (Mantel *R* = 0.63, *p* = 0.001) both exhibited strong positive correlations with the DeepARG profile. These correlations suggest that although absolute abundance estimates differ among methods, the overall trends in resistome diversity and evenness remain consistent when comparing the deep learning approach with selected alignment‐based databases. Hence, although the raw counts of predicted ARGs vary substantially, the relative rankings of samples by diversity metrics are comparable.

The observed agreement between DeepARG and PanRes diversity indices may be influenced not only by ecological patterns but also by shared database composition. Since both tools aggregate ARGs from multiple reference sources, database overlap may contribute to correlated diversity structures, complicating the interpretation of these patterns as purely biological. Also, we highlight that annotations were not harmonized to a common ontology (e.g., ARO) prior to cross‐method comparisons; therefore, differences in naming granularity may influence diversity indices and between‐method correlations. Moreover, although all contig‑based annotations in this comparison were normalized using RPKM, the read‐based approach of AMR++ uses read‑level counts, which means that abundance estimates reflect methodological differences rather than directly comparable performance metrics. Still, these outcomes reinforce the idea that the choice of methodology not only influences detection sensitivity but may also affect ecological interpretations when comparing multiple sampling sites across regional gradients.

## 2 THE ANNOTATION METHOD INFLUENCES ECOLOGICAL INTERPRETATIONS OF THE ENVIRONMENTAL RESISTOME PROFILE

Environmental associations represent an important facet of environmental resistome studies, particularly in dynamic ecosystems [5]. To explore how the choice of annotation method affected our understanding of the environmental drivers shaping the resistome, we conducted a series of analyses based on Bray–Curtis dissimilarities in ARG profiles between methods. Principal Coordinate Analysis plots revealed clear regional differentiation in ARG composition (Figures S2–S3), and a permutation test for ADONIS under a reduced model confirmed that most methods, except for ResFinder with an 80% identity threshold, yielded statistically significant differences in ARG profiles between defined regions (Figure 2A). To complement these observations, we employed distance‐based redundancy analysis (dbRDA) to quantify the influence of environmental variables (e.g., salinity, temperature, nutrient levels) on the ARG composition as determined by each method (Figure 2B and Figure S4). The dbRDA for DeepARG indicates that 24% of the variation in predicted ARG composition could be directly associated with specific environmental drivers. For comparison, AMR++ achieved 29%, while PanRes came in lower at 10%. Although AMR++ showed a higher proportion of explained variation, it detected far fewer ARG annotations than DeepARG (i.e., 1656 vs. 2042 annotations). Thus, DeepARG's higher detection rate, coupled with its robust environmental associations, provides an overview of resistome variation as captured under different annotation strategies in the Baltic Sea, enabling a more detailed comparison of how abiotic gradients are associated with predicted ARG profiles across regions. These analyses collectively indicate that the ARG composition derived from DeepARG showed a strong apparent association with regional and environmental gradients.

**Figure 2:**
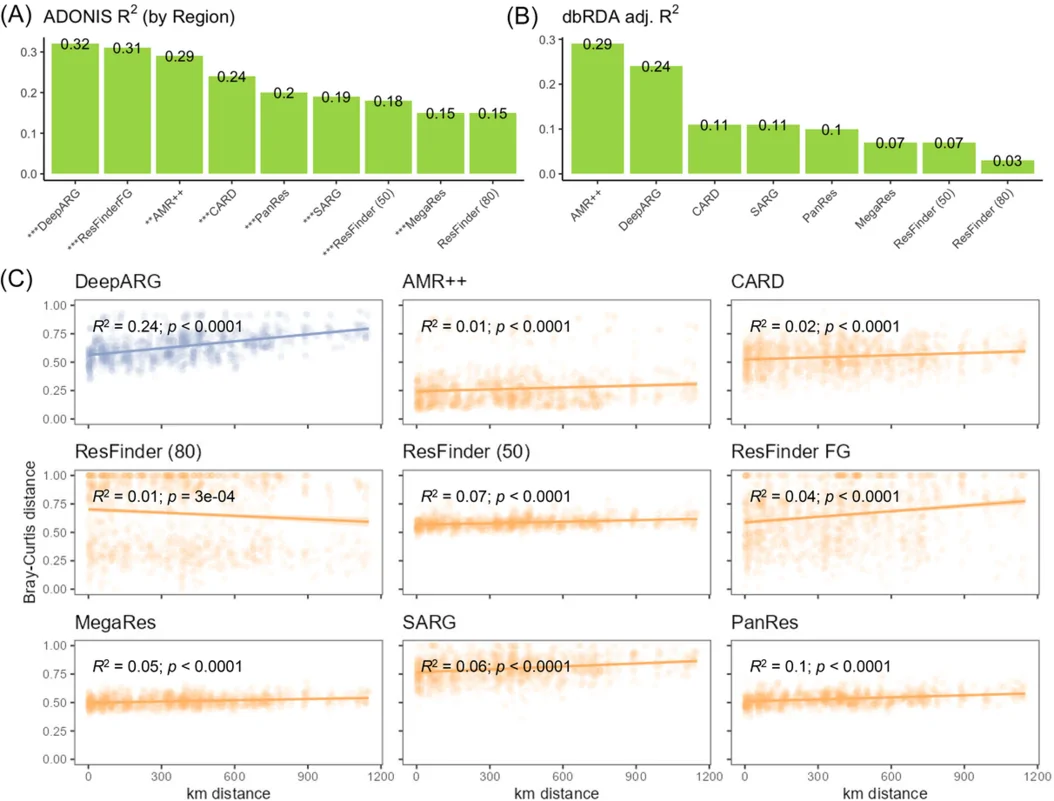
Environmental associations of the resistome profiles. (A) ADONIS *R* ^2^ based on the relative influence of region on the community dissimilarity between methods, and the (B) adjusted *R* ^2^ of the distance‐based redundancy analyses (dbRDA). The *** in the ADONIS results indicate significance at *p*‐value < 0.001, and ** at *p*‐value < 0.01 (Figure S4). (C) Distance decay relationship of the Bray–Curtis dissimilarities of the ARG annotations per method. The geographic distance (in km) was calculated using the haversine formula from the latitude and longitude coordinates of each site relative to the northernmost site. Environmental variables are listed in Table S3.

Most notably, when we assessed the distance–decay relationship (i.e., the degree to which resistome composition changes with geographical distance) across methods, geographical distance had the strongest relative influence on the DeepARG‐derived composition, accounting for 24% of the variability in Bray–Curtis dissimilarities (Figure 2C). In contrast, the variable influence of distance on the ARG composition for other methods was less than 7%. This suggests that, under native ontologies, the DeepARG‐based profile could have captured stronger spatial structuring, resolving subtle ecological gradients in environmental resistomes, which is essential in delineating how regional variations and physical separation influence ARG distribution in marine ecosystems. Still, we note that this pattern could not be interpreted as definitive evidence of stronger spatial structuring, and that the observed relationship may also reflect differences in annotation breadth, database composition, or noise amplification inherent to the tool's native ontology. Hence, the distance–decay results represent methodological contrasts rather than validated ecological signal strength.

## 3 TOWARDS CONSENSUS PIPELINES FOR ROBUST RESISTOME PROFILING FROM ENVIRONMENTAL METAGENOMES

By using a combination of comparative metrics, including diversity measures and environmental associations, we show that DeepARG yields a broader annotation space under its native configuration, facilitating comparative ecological analyses providing a deeper understanding of how environmental factors influence the distribution of ARGs in marine ecosystems [5]. However, the differences in performance among various annotation strategies suggest that a consensus or hybrid approach may ultimately provide the most reliable resistome assessments. For example, while DeepARG's deep learning framework excels in sensitivity and identifying broader resistance profiles, alignment‐based methods using well‐curated databases, i.e., CARD, ResFinder, or PanRes, offer a level of specificity that can validate the more inclusive predictions from DeepARG. The strong positive correlations among DeepARG, ResFinder (at a relaxed threshold), and PanRes for diversity indices highlight the value of combining these methods. However, because this study does not include method‑agnostic accuracy benchmarks, we cannot assess the true‐positive or false‐positive rates for any tool. Consequently, higher annotation counts, particularly for DeepARG, should not be interpreted as improved accuracy. Instead, they reflect differences in model architecture, database breadth, and sensitivity thresholds.

Environmental association analyses further indicate that, while DeepARG appears sensitive to detecting regional and environmental patterns, its results are most meaningful when supported by independent validation from alternative annotation frameworks, for example, read alignment pipelines (AMR++). In practice, this involves carefully benchmarking candidate software against datasets that closely match the complexity of the target environment. Integrating these approaches into a consensus‐driven pipeline, for example, argNorm and hAMRoaster, can help balance sensitivity and specificity, thereby improving the overall robustness of environmental resistome profiling. The present study did not include method‐agnostic accuracy benchmarks. Hence, in the absence of comprehensive environmental ground truth, we used ecological structure and diversity comparisons as complementary indicators of signal capture. Although ecological structure strength and higher counts do not necessarily indicate better accuracy, they reflect over‐detection, ontology breadth, or compositional effects on the resistome profile across methods. Our findings, therefore, reflect comparative ecological signal patterns rather than tool accuracy, and future work incorporating spike‑in or synthetic benchmarking datasets will be necessary to evaluate sensitivity and false‑positive rates. Formal accuracy benchmarking with controlled truth sets is needed to quantify sensitivity and specificity, and represents a critical next step beyond the scope of the present ecological comparison.

Lastly, the selection of bioinformatics tools for metagenomic AMR prediction must be tailored to the specific research or clinical question and to the acceptable balance between false negatives and false positives [7, 10, 12]. DeepARG represents an early‑generation convolutional neural network model that has been widely adopted as a baseline tool for metagenomic ARG prediction. However, it does not incorporate more recent advances in deep learning, for example, graph neural network architectures (e.g., ARGNet) or protein language models (e.g., ESM‐2 and PLM‐ARG), which may offer improved generalization and reduced false‑positive rates. We therefore interpret DeepARG's performance in this study as representative of a commonly used baseline rather than a state‑of‑the‑art model. Importantly, the stronger spatial pattern observed for DeepARG may arise from additional factors unrelated to ecological processes, including a broader annotation space, increased detection of low‑similarity sequences, or database‑driven biases. Because we did not perform leave‑one‑out cross‑validation or evaluate correlations with environmental versus clinical genome similarity, alternative explanations cannot be excluded. The distance‑decay analysis should therefore be interpreted with caution, and the differences in *R^2^* values across tools may reflect differences in ontology structure, annotation granularity, or noise effects rather than true ecological gradients. Future work incorporating cross‑validation or genome‑similarity analyses will be necessary to disentangle methodological artifacts from biological signal. Hence, our conclusions should be interpreted as ecosystem‑specific, and future work incorporating multiple environments will be necessary to evaluate the broader applicability of these comparative insights.

In conclusion, our report not only reinforces the utility of deep learning‐based methods for environmental applications but also provides a pathway for future methodological improvements that can bridge the gap between expansive detection and clinically relevant risk interpretation. Future strategies that integrate multiple annotation approaches may further advance our understanding of ARG dynamics in environmental systems, ultimately informing targeted interventions and public health risk assessments.

## AUTHOR CONTRIBUTIONS

**Joeselle M. Serrana**: Conceptualization; methodology; validation; data curation; formal analysis; visualization; writing—original draft. **Francisco J. A. Nascimento**: Data curation; resources; writing—review and editing. **Malte Posselt**: Writing—review and editing; supervision; resources; funding acquisition.

## CONFLICT OF INTEREST STATEMENT

The authors declare no conflicts of interest.

## ETHICS STATEMENT

No animals or humans were involved in this study.

## Supporting information


**Figure S1:** Average relative abundance of the ARG types per region and hierarchical clustering.
**Figure S2:** Principal coordinate analysis (PCoA) plot of the ARG profile based on Bray–Curtis distance grouped by Region.
**Figure S3:** Boxplot of the Bray–Curtis distance in each region.
**Figure S4:** Distance‐based redundancy analysis (dbRDA) ordination plot of the ARG composition based on Bray–Curtis distance and the log1p‐transformed environmental factors.

**Table S1:** List of antimicrobial resistance (AMR) prediction tools evaluated in this study. The relative abundance of antimicrobial resistance gene (ARG) annotations for each method and their categories is presented in Figure S1.
**Table S2:** Comparison of methods for antimicrobial resistance gene (ARG) profiling: Reads per kilobase million (RPKM) absolute counts.
**Table S3:** Metadata: Sample information and environmental factors from Serrana et al.[5].

## Data Availability

The data that support the findings of this study are openly available in GitHub at https://github.com/jserrana/baltic-amr/tree/main/MS-AMR-Database_Analysis. The Baltic Sea metagenomic sequencing data used in this study are deposited in the European Nucleotide Archive under accession number PRJEB41834 (ERR5010645‐661 and ERR5010683‐724). The metagenomics processing is described in Serrana et al. [5]. Additional information on the methods is presented in the Supplementary Material. The corresponding input data and code for visualization and statistical analysis are available at https://github.com/jserrana/baltic-amr/tree/main/MS-AMR-Database_Analysis. Supplementary materials (methods, figures, tables, scripts, graphical abstract, slides, videos, Chinese translated version, and updated materials) may be found in the online DOI or iMetaOmics Science http://www.imeta.science/imetaomics.
