# Supplementary material for: Metagenomic mining of environmental resistomes: Comparative performance of deep learning and alignment‐based annotation methods
Source: IMetaOmics. 2026 Jun 9;3(2):e70107. doi: 10.1002/imo2.70107 (PMC13674460; doi:10.1002/imo2.70107)
Supplement: Supplementary file 1 [file IMO2-3-e70107-s002.docx]

# Supporting Information to:

**Metagenomic mining of environmental resistomes: Comparative performance of deep learning and alignment-based annotation methods**

**Running title**: Metagenomic mining of environmental resistomes

Joeselle M. Serrana^1,2^*, Francisco J. A. Nascimento^3^, Malte Posselt^2^

^1^Stockholm University Center for Circular and Sustainable Systems (SUCCeSS), Stockholm University, 106 91 Stockholm, Sweden

^2^Department of Environmental Science (ACES), Stockholm University, 106 91 Stockholm, Sweden

^3^Department of Ecology, Environment, and Plant Sciences (DEEP), Stockholm University, 106 91 Stockholm, Sweden

*Correspondence: [joeselle.serrana@aces.su.se](mailto:joeselle.serrana@aces.su.se) (Joeselle M. Serrana)

## Supplementary Materials and Methods

### Sample description and metagenomics data processing

Metagenomics data from 59 benthic sediments were used in this study [1]. Bioinformatics processing are described in Serrana et al. [2]. Samples were grouped into eight regions, i.e., Bothnian Bay, Bothnian Sea, Stockholm, Sörmland, Dead Zone (Sörmland), Östergötland, Dead Zone (Mid-South), and Southern Baltic. Environmental variables used to assess the association between the ARG profile include water depth (m), salinity, temperature, and dissolved O_2_ (mg/L). The concentrations of total carbon (TC) and total nitrogen (TN) (µmol/g), and their ratio – C/N, and isotopic signatures, i.e., stable δ^13^C and δ^15^N isotope compositions (‰).

### Prediction of antimicrobial resistance genes

Quality-filtered reads, and contigs were annotated using different computational approaches and against different ARG databases. Our approach involved two parallel strategies for annotation. For the deep learning-based method, we used the DeepARG (LS model) to perform ARG annotations on assembled contigs derived from metagenomic data [3]. In contrast, for the alignment-based method, quality-filtered paired-end reads were annotated using the AMR++ workflow [4], and contigs were matched against CARD [5], ResFinder [6], ResFinder FG [7], MegaRes [13], SARG [8], and PanRes [9] applying defined thresholds (80% identity and 70% query coverage, with a supplemental assessment for ResFinder at a relaxed 50% identity threshold). Predicted ARG abundances were expressed as reads per kilobase million (RPKM) for each database to allow for a systematic performance comparison (**Table S2**). RPKM was used to normalize gene coverage values by correcting the differences in sample sequencing depth and gene length. The RPKM value of the gene is calculated using the equation [10]:

$$RPKM=\frac{numReads}{\left( \frac{geneLength}{{10}^{3}} \right) \times\left( \frac{totalNumReads}{{10}^{6}} \right)}$$

where $numReads$ is the number of reads mapped to a gene sequence, $geneLength$ is the length of the gene sequence, and $totalNumReads$ is the total number of mapped reads of a sample. The relative ARG abundance across samples was visualized by summing the RPKM for all ARGs belonging to a type (AMR category) or a subtype (gene level).

### Statistical analysis

The statistical analyses and visualizations were performed in the R environment v4.4.2 [11]. The microeco v1.13.0 [12] package was used to calculate the alpha and beta diversities of each dataset. The alpha diversity indices assessed were Chao1 richness, Shannon–Wiener diversity, and Pielou’s evenness. The abundance-based Bray-Curtis dissimilarity index was used to assess beta diversity. Principal coordinate analysis (PCoA) was performed to visualize the Bray-Curtis dissimilarity matrices. To assess the significant differences in alpha and beta diversity metrics between regions, a one-way analysis of variance (ANOVA) with Duncan's multiple comparison tests or the Dunn's Kruskal-Wallis (KW) multiple comparisons test was used. Distance decay was computed as the slope of the ordinary least-squares regression line fitted to the relationship between the haversine (km) distance and the Bray-Curtis dissimilarity distance. The distance decay rate of each dataset was plotted using the plot_scatterfit function of the microeco package.

Mantel tests and Procrustes analysis were used to evaluate the congruency between the DeepARG-annotations and the other ARG datasets using Bray-Curtis dissimilarity matrices with the vegan package. Mantel tests were performed using 9,999 permutations with Pearson correlations between distance matrices. Procrustes analyses were conducted using the procrustes() and protest() functions with 9,999 permutations to assess concordance between ordinations. The association between environmental factors (**Table S3**) and predicted ARG composition was assessed using distance-based redundancy analysis (dbRDA) using the trans_env class in the microeco package. ADONIS (PERMANOVA) tests were run using the adonis2() function with 9,999 permutations under a reduced model to evaluate the effect of region on community composition. Distance‑based redundancy analysis (dbRDA) was performed using capscale() with Bray–Curtis distances and significance assessed via permutation tests (9,999 permutations). Environmental variables were standardized prior to dbRDA to ensure comparability.

## Supplementary Figures

#### Figure S1 (1/3). Antimicrobial Resistance Gene (ARGs) composition per tool/database. Relative abundance and compositional structure of antibiotic resistance genes (ARGs) across Baltic Sea sampling sites. Stacked bars show the mean average contributions of ARG classes per region. Clustering is performed with the McQuitty hierarchical clustering algorithm. ARG categories reflect each tool’s native ontology and ortholog definitions, meaning class labels and granularity differ across databases.

 Figure S1 (2/3). **Antimicrobial Resistance Gene (ARGs) composition per tool/database.** Relative abundance and compositional structure of antibiotic resistance genes (ARGs) across Baltic Sea sampling sites. Stacked bars show the mean average contributions of ARG classes per region. Clustering is performed with the McQuitty hierarchical clustering algorithm. ARG categories reflect each tool’s native ontology and ortholog definitions, meaning class labels and granularity differ across databases.

 Figure S1 (3/3). **Antimicrobial Resistance Gene (ARGs) composition per tool/database.** Relative abundance and compositional structure of antibiotic resistance genes (ARGs) across Baltic Sea sampling sites. Stacked bars show the mean average contributions of ARG classes per region. Clustering is performed with the McQuitty hierarchical clustering algorithm. ARG categories reflect each tool’s native ontology and ortholog definitions, meaning class labels and granularity differ across databases.

### Figure S2. Beta diversity of resistome composition across Baltic Sea regions. Principal coordinate analyses (PCoA) based on the nine annotation frameworks: DeepARG, AMR++, CARD, ResFinder (80), ResFinder (50), ResFinder FG, MegaRes, SARG, and PanRes. Polygons delineate regional groupings, and axes show variance explained by PCo1 and PCo2. ARG categories reflect each tool’s native ontology and ortholog structure, resulting in differences in clustering strength and variance partitioning. PERMANOVA (adonis) R² and p‑values indicate how strongly geographic region explains resistome variation, with higher R² values (e.g., DeepARG, AMR++, ResFinder FG) showing clearer regional structuring than lower‑resolution or broader‑scope databases.

### Figure S3. Bray-Curtis dissimilarity of resistome composition across Baltic Sea regions. Boxplots show pairwise Bray–Curtis distances among sites within each region, derived from nine annotation frameworks: DeepARG, AMR++, CARD, ResFinder (80), ResFinder (50), ResFinder FG, MegaRes, SARG, and PanRes, with letters denoting significant differences (Tukey’s HSD, p < 0.05). ARG categories correspond to each database’s native ontology and ortholog definitions, influencing observed variability and clustering strength. Higher median dissimilarities in dead‑zone and southern basins indicate greater heterogeneity in ARG composition, consistent with environmental gradients and oxygenation status.

#### Figure S4. Environmental structuring of resistomes across nine ARG annotation frameworks. Distance‑based redundancy analyses (dbRDA) showing how environmental gradients structure resistome composition across Baltic Sea regions for nine annotation frameworks: DeepARG, AMR++, CARD, ResFinder (80), ResFinder (50), ResFinder FG, MegaRes, SARG, and PanRes. Points represent samples colored by region, while black vectors indicate significant environmental predictors (salinity, temperature, depth, dissolved O₂, C:N ratio, δ¹³C, and δ¹⁵N). Axes show variance explained by dbRDA1 and dbRDA2. ARG categories follow each database’s native ontology and ortholog definitions, influencing the strength of environmental associations. Adjusted R² values summarize model explanatory power, with higher values (e.g., ResFinder FG, SARG, AMR++) indicating stronger coupling between environmental gradients and resistome structure.

# REFERENCES

1. Broman, Elias, Dandan Izabel-Shen, Alejandro Rodriguez-Gijon, Stefano Bonaglia, Sarahi L. Garcia, and Francisco JA Nascimento. 2022. "Microbial functional genes are driven by gradients in sediment stoichiometry, oxygen, and salinity across the Baltic benthic ecosystem." *Microbiome* 10: 126. <https://doi.org/10.1186/s40168-022-01321-z>
2. Serrana, Joeselle M., Francisco J. A. Nascimento, Benoît Dessirier, Elias Broman, and Malte Posselt. 2025. "Environmental drivers of the resistome across the Baltic Sea." *Microbiome* 13: 92. <https://doi.org/10.1186/s40168-025-02086-x>
3. Arango-Argoty, Gustavo, Emily Garner, Amy Pruden, Lenwood S. Heath, Peter Vikesland, and Liqing Zhang. 2018. "DeepARG: a deep learning approach for predicting antibiotic resistance genes from metagenomic data." *Microbiome* 6: 23. <https://doi.org/10.1186/s40168-018-0401-z>
4. Bonin, Nathalie, Enrique Doster, Hannah Worley, Lee J. Pinnell, Jonathan E. Bravo, Peter Ferm, Simone Marini, et al. 2023. “MEGARes and AMR++, v3.0: an updated comprehensive database of antimicrobial resistance determinants and an improved software pipeline for classification using high‑throughput sequencing.” *Nucleic Acids Research* 51: D744–D752. <https://doi.org/10.1093/nar/gkac1047>
5. Alcock, Brian P., Amogelang R. Raphenya, Tammy TY Lau, Kara K. Tsang, Mégane Bouchard, Arman Edalatmand, William Huynh, et al. 2020. “CARD 2020: antibiotic resistome surveillance with the comprehensive antibiotic resistance database.” *Nucleic Acids Research* 48: D517–D525. <https://doi.org/10.1093/nar/gkz935>
6. Bortolaia, Valeria, Rolf S. Kaas, Etienne Ruppe, Marilyn C. Roberts, Stefan Schwarz, Vincent Cattoir, Alain Philippon, et al. 2020. “ResFinder 4.0 for predictions of phenotypes from genotypes.” *Journal of Antimicrobial Chemotherapy* 75: 3491–3500. <https://doi.org/10.1093/jac/dkaa345>
7. Gschwind, Rémi, Svetlana Ugarcina Perovic, Maja Weiss, Marie Petitjean, Julie Lao, Luis Pedro Coelho, and Etienne Ruppé. 2023. “ResFinderFG v2.0: a database of antibiotic resistance genes obtained by functional metagenomics.” *Nucleic Acids Research* 51: W493–W500. <https://doi.org/10.1093/nar/gkad384>
8. Yin, Xiaole, Xiao-Tao Jiang, Benli Chai, Liguan Li, Ying Yang, James R. Cole, James M. Tiedje, and Tong Zhang. 2018. “ARGs‑OAP v2.0 with an expanded SARG database and Hidden Markov Models for enhancement characterization and quantification of antibiotic resistance genes in environmental metagenomes.” *Bioinformatics* 34: 2263–2270. <https://doi.org/10.1093/bioinformatics/bty053>
9. Martiny, Hannah-Marie, Nikiforos Pyrounakis, Thomas N. Petersen, Oksana Lukjančenko, Frank M. Aarestrup, Philip TLC Clausen, and Patrick Munk. 2024. “ARGprofiler—a pipeline for large‑scale analysis of antimicrobial resistance genes and their flanking regions in metagenomic datasets.” *Bioinformatics* 40: btae086. <https://doi.org/10.1093/bioinformatics/btae086>
10. Mortazavi, Ali, Brian A. Williams, Kenneth McCue, Lorian Schaeffer, and Barbara Wold. 2008. "Mapping and quantifying mammalian transcriptomes by RNA-Seq." *Nature Methods* 5: 621–628. <https://doi.org/10.1038/nmeth.1226>
11. Team, R. Core. 2025. "RA language and environment for statistical computing, R Foundation for Statistical." Computing.
12. Liu, Chi, Yaoming Cui, Xiangzhen Li, and Minjie Yao. 2021. "microeco: an R package for data mining in microbial community ecology." *FEMS Microbiology Ecology* 97: fiaa255. <https://doi.org/10.1093/femsec/fiaa255>
